# Facile synthesis of ordered mesoporous zinc alumina catalysts and their dehydrogenation behavior†

**DOI:** 10.1039/c9ra00217k

**Published:** 2019-03-28

**Authors:** Ming Cheng, Huahua Zhao, Jian Yang, Jun Zhao, Liang Yan, Huanling Song, Lingjun Chou

**Affiliations:** State Key Laboratory for Oxo Synthesis and Selective Oxidation, Lanzhou Institute of Chemical Physics (LICP), Chinese Academy of Sciences Lanzhou 730000 PR China ljchou@licp.cas.cn songhl@licp.cas.cn; University of Chinese Academy of Sciences Beijing 100049 PR China; Suzhou Research Institute of LICP, Chinese Academy of Sciences Suzhou 215123 PR China

## Abstract

Ordered mesoporous Zn/Al_2_O_3_ materials with varying Zn content were simply prepared *via* an evaporation-induced self-assembly (EISA) method. Dehydrogenation of isobutane to isobutene was carried out on these materials; an isobutane conversion of 45.0% and isobutene yield of 39.0% were obtained over the 10%Zn/Al_2_O_3_ catalyst at 580 °C with 300 h^−1^ GHSV. The obtained materials with Zn content up to 10% possess large specific surface area and big pore volume and zinc species can be highly dispersed on the surface or incorporated into the framework. The acidity of these catalysts was changed by the introduction of Zn, the decrease of strong acid sites is conducive to the promotion of isobutene selectivity and the weak and medium acidic sites played an important role in isobutane conversion. In addition, these catalysts exhibited good catalytic stability, due to the effective inhibition of coke formation by the ordered mesoporous structure.

## Abstract 2

Facile synthesis of ordered mesoporous zinc alumina catalysts that exhibited great catalytic activity (45.0% isobutane conversion, 86.7% isobutene selectivity) and stability.

## Graphical abstract

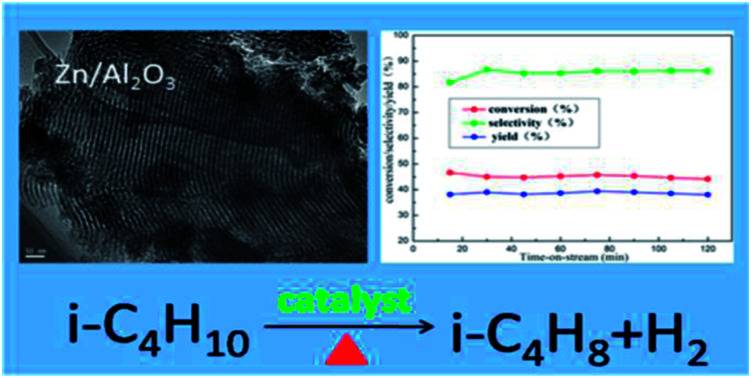

## 1. Introduction

Isobutene is one of the most important raw materials and intermediates to produce butyl rubber, ETBE (ethyl *tert*-butyl ether), polyisobutene and other chemicals.^1,2^ To better fulfill the ever-increasing market demand, a small portion of its production is by isobutane dehydrogenation.^3^ Currently, Cr_2_O_3_/Al_2_O_3_ and Pt/Al_2_O_3_ are the two most common and employable catalysts in industrial production.^4,5^ However, some unavoidable drawbacks limit their further application, because Pt is expensive and has poor availability, while Cr is not environmentally-friendly due to its toxicity. Therefore, the exploitation of novel catalysts with superior catalytic properties, low cost and non-pollution for isobutane dehydrogenation is highly recommendable.^6–8^

At present, all kinds of metal catalysts, such as vanadium-based, iron-based, molybdenum-based catalysts, have been widely investigated in this dehydrogenation reaction.^9–12^ In particular, owing to the excellent catalytic performance, Zn based catalysts have been interested and gazed considerably in the direct dehydrogenation of isobutane. For example, Zn modified HZSM-5 materials were found effective for activation of isobutane and high conversion obtained.^13,14^ Unfortunately, the poor selectivity with respect to the formation of many undesired dry gas (CH_4_ and C_2_H_6_) impeded its more broad applications, which probably derived from redundant strong acidic sites of HZSM-5 zeolite. Liu *et al.* reported that 27.5% isobutane conversion and 83.8% isobutene selectivity obtained in isobutane dehydrogenation over Zn/S-1 catalyst, because strong acid sites were absent.^15^ Moreover, Zn/Ti thin film and Zn/Ga oxide catalyst has also been referred in isobutane dehydrogenation.^16,17^

Recently, ordered and adjustable mesoporous alumina materials have drawn research much attention. Coupled with their moderate surface acidity and good thermostability, ordered mesoporous alumina as heterogeneous catalyst support has a wonderful applying prospect.^18^ Morris *et al.* successfully synthesized NiO–Al_2_O_3_ composites with ordered mesoporous structure and the metal oxide well dispersed on the alumina support.^19^ Yuan *et al.* reported a simple route to synthesize γ-Al_2_O_3_ using P123 template agent, which have high quality mesoporous structure.^20^ Schweitzer *et al.* put forward the computed minority catalytic pathway consists of undesired C–C bond cleavage at Zn(ii) site had a significantly higher activation energy barrier and the high olefin selectivity observed for single-site Zn(ii) on SiO_2_.^21^ In our previous work, well-ordered mesoporous Cr_2_O_3_/Al_2_O_3_ catalysts were synthesized and showed an advantage in catalytic stability.^22^ So far, almost no research was reported on ordered mesoporous zinc alumina composites for alkane dehydrogenation.

This study firstly prepared ordered mesoporous *x*Zn/Al_2_O_3_ catalysts with different Zn content *via* evaporation-induced self-assembly (EISA) method and evaluated the catalytic performances in isobutane dehydrogenation. The textural properties of obtained materials were characterized by XRD analysis, N_2_ adsorption–desorption and TEM. And we also discussed the form of Zn species, metal-support interaction and surface acidity by XPS, H_2_-TPR and NH_3_-TPD. Besides, all the characterizations were associated with catalytic reactions.

## 2. Experimental

### 2.1. Catalyst preparation

Ordered mesoporous Zn/Al_2_O_3_ materials were prepared *via* EISA method according to the previous literatures.^20,23,24^ In a typical procedure, 1.0 g P123 as surfactant was added to 20 ml ethanol and the solution was stirred for 40 min, followed by the addition of a mmol aluminum isopropoxide, *b* mmol Zn(NO_3_)_2_·6H_2_O (*a* + *b* = 10 mmol) and 1.6 ml nitric acid. After 6 h of stirring, the mixed solution was transferred to a culture dish and kept in drying oven at 60 °C for two days. The obtained xerogel was calcined at 600 °C for 5 h. The catalysts were denoted as *x*Zn/Al_2_O_3_, where the nominal molar ratio *x* = (*b*/(*a* + *b*) × 100%).

### 2.2. Catalytic dehydrogenation

The direct dehydrogenation of isobutane was studied in fixed-bed quartz reactor. Typically, 900 mg of catalyst was sieved at 60–80 mesh. The reaction temperature was set at 560–620 °C. The reactant gas was fed by gas hourly space velocity (GHSV) of 300 h^−1^. The composition of the gaseous products was analyzed on-line using gas chromatography fitted with flame ionization detector (FID) and thermal conductivity detector (TCD).

### 2.3. Characterization

N_2_ adsorption–desorption isotherms were measured by Autosorb-iQ analyzer. The specific surface area was calculated by Brunauer–Emmett–Teller (BET) equation and pore size distribution was calculated by Barret–Joyner–Halenda (BJH) equation from the N_2_ sorption isotherm.

X-ray diffraction (XRD) were collected on X'Pert Pro diffraction instrument using Cu Kα radiation over the range 0.6° < 2*θ* < 5° and 5° < 2*θ* < 80°.

Transmission electron microscopy (TEM) images were conducted at TECNAIG^2^ F20 instrument.

X-ray photoelectron spectroscopy (XPS) were recorded on Thermon ESCALAB 250xi spectrometer. The binding energies were calibrated against at 284.8 eV of C1s.

H_2_ temperature-programmed reduction (H_2_-TPR) was performed on ChemBET Pulsar Analyzer. Prior to the tests, sample was pretreated at 300 °C in He flow for 30 min. After cooling to ambient temperature, then raised temperature to 650 °C in 10% H_2_–Ar mixed flow.

NH_3_ temperature-programmed desorption (NH_3_-TPD) was recorded on ChemBET Pulsar analyzer combining with mass spectroscopy. After being pretreated at 300 °C in He flow, the sample adsorbed NH_3_ to saturation at 120 °C. The profile was detected from 50 °C to 600 °C.

Thermogravimetric-differential scanning calorimetry (TG-DSC) was based on a NETZSCH STA 449F3 analyzer under air within 20–800 °C at 10 °C min^−1^.

The Zn content in a series of *x*Zn/Al_2_O_3_ catalysts were measured by inductively coupled plasma optical emission spectrometer (ICP-OES, 725-ES, Agilent).

## 3. Results and discussion

### 3.1. XRD analysis

**Fig. 1:**
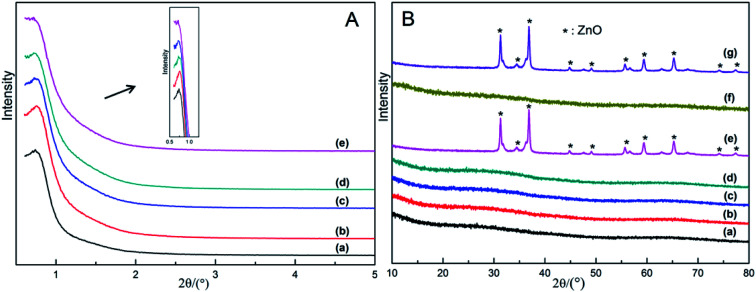
The XRD patterns of the as-synthesized and spent *x*Zn/Al_2_O_3_ catalysts: (a) 3%Zn/Al_2_O_3_; (b) 5%Zn/Al_2_O_3_; (c) 7%Zn/Al_2_O_3_; (d) 10%Zn/Al_2_O_3_; (e) 15%Zn/Al_2_O_3_; (f) the spent 10%Zn/Al_2_O_3_; (g) the spent 15%Zn/Al_2_O_3_.

The XRD patterns of all the as-synthesized *x*Zn/Al_2_O_3_ materials are presented in Fig. 1. As displayed from Fig. 1A inside, a distinct reflection peak was observed in 3–10%Zn/Al_2_O_3_ samples at the characteristic reflection (100) of *p*6*mm* space group, which confirming that these materials were composed of a well ordered mesoporous structure.^25^ With further increasing Zn content to 15%, the diffraction peak at 0.8° became almost disappeared, indicating the significant effect of zinc content in building the ordered mesoporous structure. Part B of Fig. 1 presents the wide-angle XRD patterns of all the materials, which revealed the existence of amorphous Al_2_O_3_. Only the 15%Zn/Al_2_O_3_ sample exhibited sharp diffraction peaks associated with hexagonal crystalline ZnO (no. 89-1397 from the ICDD). The average size of these ZnO particles is 23.8 nm. On the contrary, no diffraction peaks for Zn species came into sight in 3–10%Zn/Al_2_O_3_ materials with ordered mesopore, implying Zn species highly dispersed on support surface or incorporated into amorphous alumina framework.^26^ In conclusion, the presence of ordered mesoporous structure played an important role in promoting the dispersion of Zn species on the catalyst support.

### 3.2. Nitrogen adsorption–desorption analysis

**Fig. 2:**
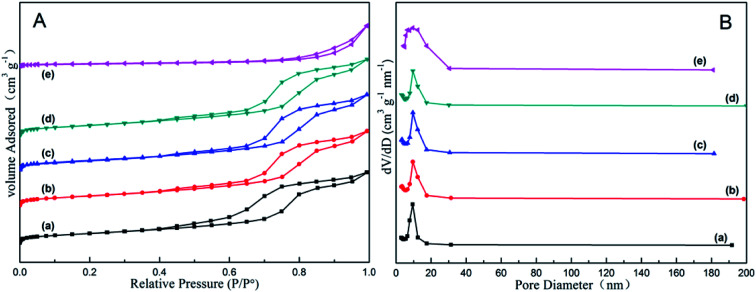
The nitrogen adsorption–desorption analysis of the as-synthesized *x*Zn/Al_2_O_3_ catalysts: (a) 3%Zn/Al_2_O_3_; (b) 5%Zn/Al_2_O_3_; (c) 7%Zn/Al_2_O_3_; (d) 10%Zn/Al_2_O_3_; (e) 15%Zn/Al_2_O_3_.

The nitrogen adsorption–desorption analysis of all the as-synthesized *x*Zn/Al_2_O_3_ materials is displayed in Fig. 2. Each sample but 15%Zn/Al_2_O_3_ exhibited typical IV type isotherm as well as H1 shaped hysteresis loop, implying the presence of uniform cylindrical mesoporous channel in these catalysts.^27^ Furthermore, the 3–10%Zn/Al_2_O_3_ samples possessed quite narrow pore size distribution around 9.5 nm, while that of 15%Zn/Al_2_O_3_ sample was bigger (around 12.4 nm). It suggested that the larger pores could be accumulated by ZnO particles. Table 1 listed the detailed data regarding to the textural properties of above samples. It has been observed that the specific surface area of as-synthesized 3–10%Zn/Al_2_O_3_ materials was similar (around 163 m^2^ g^−1^), which because Zn species highly dispersed on support surface or incorporated into alumina framework wouldn't lead to pore plugging. At a Zn content of 15%, the specific surface area was only 31 m^2^ g^−1^ owing to the mesoporous structure collapsed. It has been known that a larger surface area conduced to the better dispersion of active species, thereby can provide more “accessible” active centers for the reactant gas.^28^ Therefore, these as-synthesized 3–10%Zn/Al_2_O_3_ catalysts may own preferable catalytic performance.

**Table 1:** The textural data of the as-synthesized *x*Zn/Al_2_O_3_ catalysts^a^

Samples	*S*_BET_ (m^2^ g^−1^)	*V*_P_ (cm^3^ g^−1^)	*d*_P_ (nm)	Isotherm type	Zn content (%)	
					XPS	ICP-OES
3%Zn/Al_2_O_3_	163.3	0.41	9.5	IV	1.6	2.5
5%Zn/Al_2_O_3_	162.6	0.43	9.6	IV	2.3	4.3
7%Zn/Al_2_O_3_	163.4	0.44	9.6	IV	3.0	6.4
10%Zn/Al_2_O_3_	163.3	0.43	9.6	IV	3.8	9.7
15%Zn/Al_2_O_3_	31.3	0.23	12.4	III	14.6	12.8
10%Zn/Al_2_O_3_^a^	149.7	0.40	9.3	IV	—	—
10%Zn/Al_2_O_3_^b^	158.6	0.42	9.5	IV	—	—
15%Zn/Al_2_O_3_^a^	25.2	0.16	11.8	III	—	—

a ^a,b^Stand for the corresponding spent and fifth regenerated catalyst, respectively.

### 3.3. TEM analysis

**Fig. 3:**
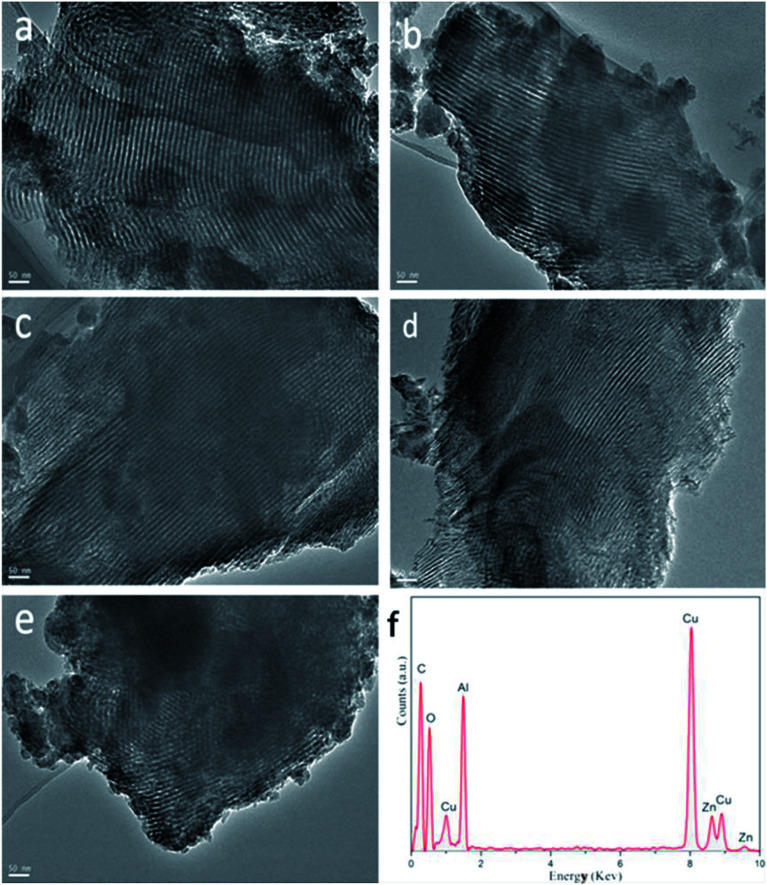
The TEM images of the as-synthesized *x*Zn/Al_2_O_3_ catalysts: (a) 3%Zn/Al_2_O_3_; (b) 5%Zn/Al_2_O_3_; (c) 7%Zn/Al_2_O_3_; (d) 10%Zn/Al_2_O_3_; (e) 15%Zn/Al_2_O_3_. (f) EDX characterization for 10%Zn/Al_2_O_3_.

**Fig. 4:**
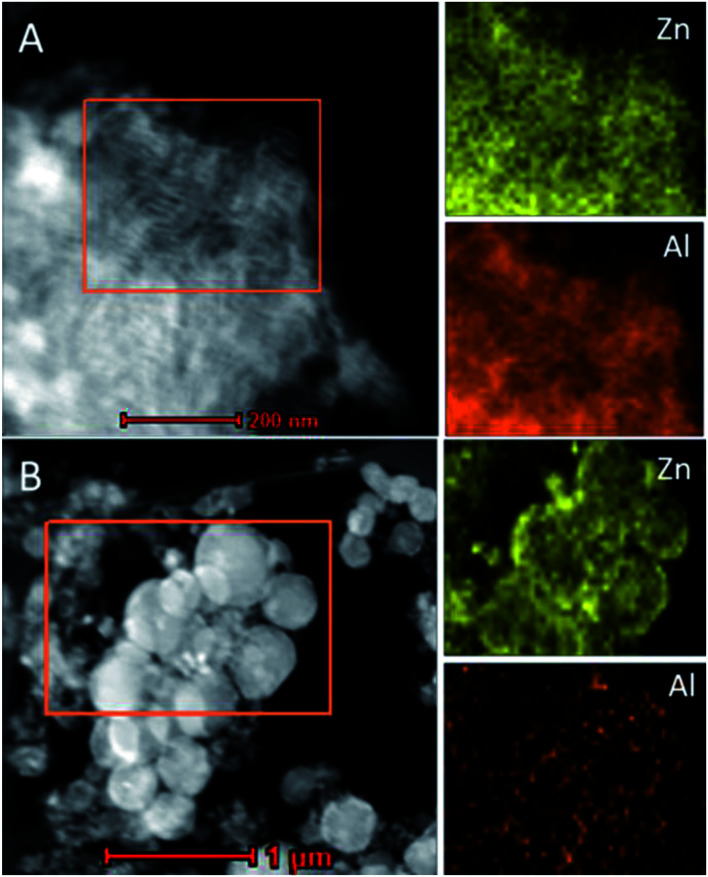
TEM image and Zn, Al elemental mappings of the fresh samples: (A) 10%Zn/Al_2_O_3_; (B) 15%Zn/Al_2_O_3_.

The morphology and structure of all the as-synthesized *x*Zn/Al_2_O_3_ materials were performed by TEM (Fig. 3). From the images, all the sample except 15%Zn/Al_2_O_3_ presented one dimensional parallel channel along [1 1 0], which intuitively confirmed the presence of well-ordered mesoporous structure in the 3–10%Zn/Al_2_O_3_ catalysts. Notably, no distinct ZnO clusters were invisible in the images, illustrating the high dispersion of Zn species on the ordered mesoporous channel. This result conformed to the low-angle XRD analysis. Only a fraction of ordered mesopore was formed for 15%Zn/Al_2_O_3_ sample. In the EDX profile of 10%Zn/Al_2_O_3_ sample (Fig. 3f), the characteristic peaks of Al, Zn, O element can be observed clearly, which verified that Zn species had been successfully loaded. Besides, elemental mapping has further confirmed the ordered mesoporous structure and shown Zn species on the surface to be highly dispersed and distributed homogeneously over the 10%Zn/Al_2_O_3_ catalyst. However, an obvious aggregation of ZnO particles was visible on the surface of 15%Zn/Al_2_O_3_ catalyst, which conformed the result of wide-angle XRD analysis (Fig. 4).

### 3.4. XPS analysis

**Fig. 5:**
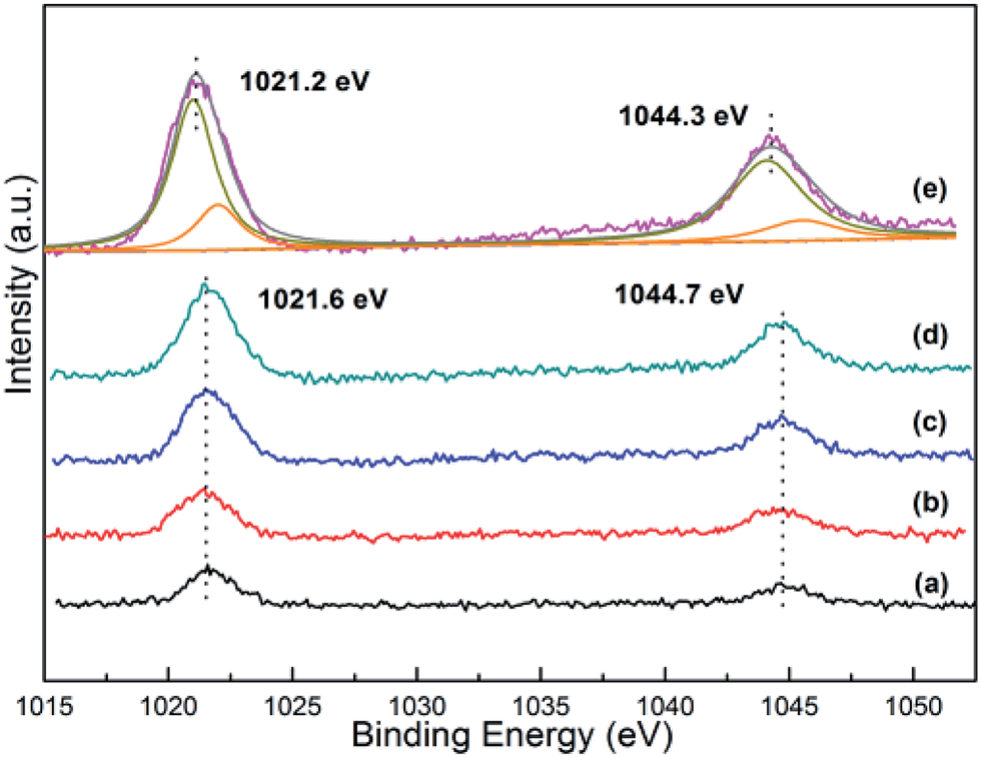
The Zn 2p XPS spectra of the as-synthesized *x*Zn/Al_2_O_3_ catalysts: (a) 3%Zn/Al_2_O_3_; (b) 5%Zn/Al_2_O_3_; (c) 7%Zn/Al_2_O_3_; (d) 10%Zn/Al_2_O_3_; (e) 15%Zn/Al_2_O_3_.

The XPS spectra of Zn 2p orbital for all the as-synthesized *x*Zn/Al_2_O_3_ materials are depicted in Fig. 5. As displayed, the binding energy located at 1021.2–1201.6 eV and 1044.3–1044.7 eV was assigned to Zn^2+^, as proved by spin–orbital splitting of 23.1 eV between Zn 2p_3/2_ and Zn 2p_1/2_.^29,30^ Noteworthy, the binding energy of Zn 2p in the sample 15%Zn/Al_2_O_3_ was lower than that of 10%Zn/Al_2_O_3_, which may be derived from the difference of Zn species in these catalysts. By deconvolution, the peak of 15%Zn/Al_2_O_3_ was divided into two peaks, the big one may be assigned to bulk ZnO particle on the surface, another small one may belonged to Zn species incorporated into alumina framework. The Zn content calculated by XPS and ICP-OES was used to affirmed and the corresponding data listed in Table 1. We can see that the bulk Zn contents were closely to the calculated data, only 15%Zn/Al_2_O_3_ had a larger deviation. Moreover, the surface Zn content (14.6%) of this sample from XPS was higher than the actual Zn content (12.8%), suggesting aggregation of ZnO particles on the surface. In contrast, the surface Zn was less than the bulk for 3–10%Zn/Al_2_O_3_ samples, which was due to most of Zn species incorporated into alumina framework. These results were accorded with wide-angle XRD analysis and confirmed the change of Zn species when increased Zn content from 10% to 15%. Besides, the higher binding energy meaning a stronger interaction between Zn species and support in 3–10%Zn/Al_2_O_3_ catalysts. This result will be further confirmed by following H_2_-TPR characterization.

### 3.5. H_2_-TPR profiles

**Fig. 6:**
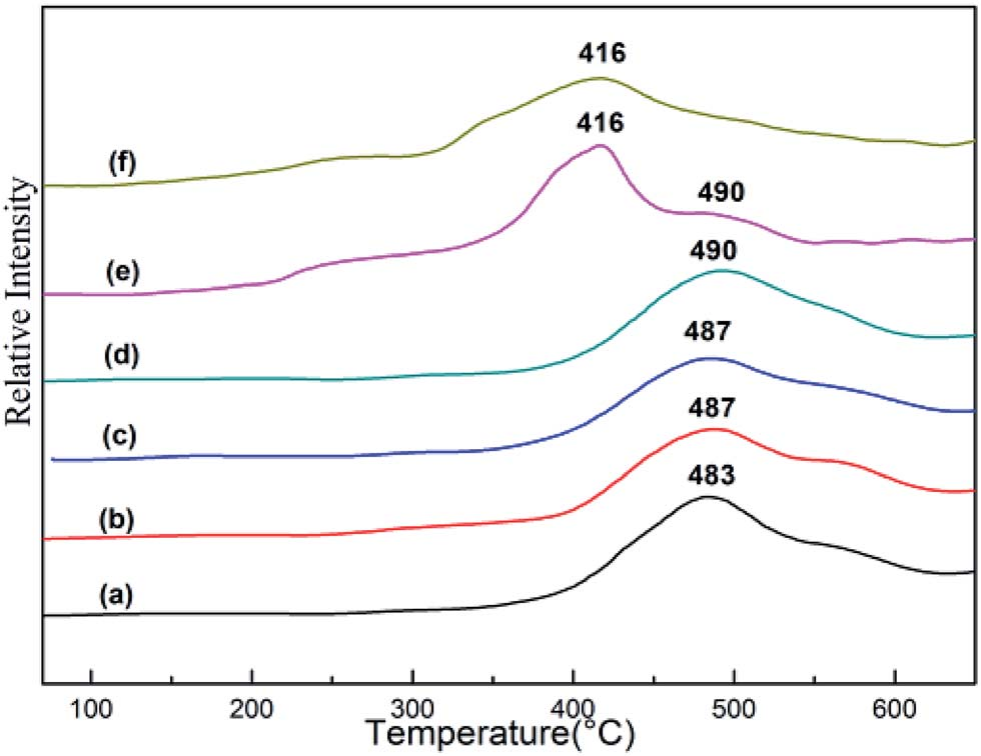
The H_2_-TPR profiles of the as-synthesized *x*Zn/Al_2_O_3_ catalysts: (a) 3%Zn/Al_2_O_3_; (b) 5%Zn/Al_2_O_3_; (c) 7%Zn/Al_2_O_3_; (d) 10%Zn/Al_2_O_3_; (e) 15%Zn/Al_2_O_3_; (f) ZnO.

H_2_-TPR characterization is a very useful instrument to investigate the interaction between Zn species and catalyst support. The results for the as-synthesized *x*Zn/Al_2_O_3_ materials and pure ZnO are shown in Fig. 6. All profiles of *x*Zn/Al_2_O_3_ showed a broad reduction peak around 490 °C, which could be assigned to the reduction of highly dispersed zinc species on support surface and Zn species incorporated into alumina framework. However, 15%Zn/Al_2_O_3_ catalyst presented a unique reduction peak at 416 °C, which was similar with the reduction peak of pure bulk ZnO. More specifically, this peak belonged to the reduction of binuclear (Zn–O–Zn)^2+^ clusters.^31^ It has been known that the higher reduction temperature, the stronger interaction between metal and support. Therefore, the Zn species with the reduction peak around 490 °C have stronger interaction with support.^32^ The characterization of H_2_-TPR was coincided with the above XRD and XPS analysis.

### 3.6. NH_3_-TPD analysis

The acidic property of all the as-synthesized *x*Zn/Al_2_O_3_ materials was determined by NH_3_-TPD and the corresponding results are displayed in Fig. S1.† All the samples exhibited a broad peak at 50–600 °C, indicating abundant different intensities of acidic sites in *x*Zn/Al_2_O_3_ catalysts. By deconvolution, the broad peak was divided into four peaks accredited to physically adsorbed NH_3_, weak, medium and strong acidic sites respectively.^33,34^ The detailed calculation result was in Table 2. As displayed, all the samples except for 15%Zn/Al_2_O_3_ exhibited similar amount of physically adsorbed NH_3_, which due to their comparable pore property. However, the specific surface area and pore volume of 15%Zn/Al_2_O_3_ was much smaller, then physically adsorbed NH_3_ also less than other samples. Besides, we can see that there are three different acidic sites on pure ordered mesoporous Al_2_O_3_. With the introduction of 3%Zn, the amount of three acidic sites significantly enhanced. It indicated that a part of three types acid sites originated from surface hydroxyl group and coordinative unsaturated Al sites on Al_2_O_3_ support, and other derived from Zn species.^15,35^ Note that the total and weak acidic sites gradually increased, while the strong acidic sites decreased slowly with the increase of Zn content until 10%. When the Zn content reached 15%, the total acidic strength of this catalyst declined sharply, which may due to bulk ZnO has rarely acidic sites (Fig. S2†). It was well established that weak and medium acid sites played a key role to isobutane conversion in dehydrogenation reaction.^15^ However, side reactions (polymerization, isomerization and cracking) are mainly catalyzed by strong acid sites.^36^ As a conclusion, the various *x*Zn/Al_2_O_3_ catalysts with the different number of acidic sites may present distinct catalytic performance.

**Table 2:** The acidic properties of the as-synthesized Al_2_O_3_ and *x*Zn/Al_2_O_3_ catalysts

Samples	Physically adsorbed NH_3_ (mmol g^−1^)	The amount of acidic sites (mmol g^−1^)			
		Weak	Medium	Strong	Total
Al_2_O_3_	0.007	0.037	0.021	0.009	0.067
3%Zn/Al_2_O_3_	0.008	0.048	0.051	0.026	0.125
5%Zn/Al_2_O_3_	0.009	0.061	0.043	0.024	0.128
7%Zn/Al_2_O_3_	0.010	0.066	0.041	0.023	0.130
10%Zn/Al_2_O_3_	0.009	0.069	0.048	0.016	0.133
15%Zn/Al_2_O_3_	0.004	0.036	0.033	—	0.069

### 3.7. Catalytic performance in the isobutane dehydrogenation

**Fig. 7:**
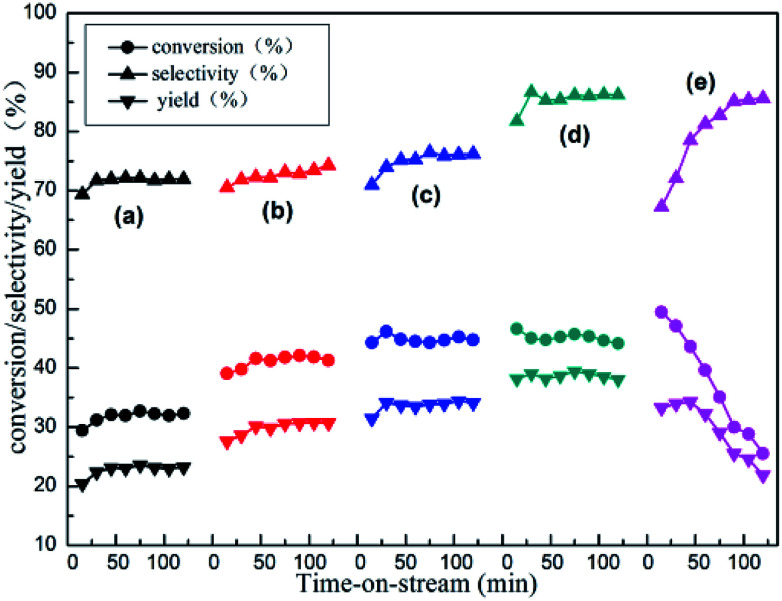
The reactivity of isobutane dehydrogenation over the as-synthesized *x*Zn/Al_2_O_3_ catalysts: (a) 3%Zn/Al_2_O_3_; (b) 5%Zn/Al_2_O_3_; (c) 7%Zn/Al_2_O_3_; (d) 10%Zn/Al_2_O_3_; (e) 15%Zn/Al_2_O_3_. Reaction condition: *T* = 580 °C, GHSV = 300 h^−1^.

The reactivity of isobutane dehydrogenation over a series of *x*Zn/Al_2_O_3_ catalysts is displayed in Fig. 7. As we can notice that the catalytic activity of *x*Zn/Al_2_O_3_ catalysts gradually increased until 10% Zn content. The 10%Zn/Al_2_O_3_ catalyst exhibited a notably higher initial isobutane conversion (46.6%) and initial isobutene selectivity (81.8%) by contrast with the 3%Zn/Al_2_O_3_ (29.4% conversion and 69.4% selectivity), indicating that Zn content was a very significant factor in isobutane dehydrogenation reaction. In addition to this, with increasing reaction time, the reactivity over the 3–10%Zn/Al_2_O_3_ catalysts can basically hold steady, while the 15%Zn/Al_2_O_3_ catalyst presented poor catalytic stability.

**Fig. 8:**
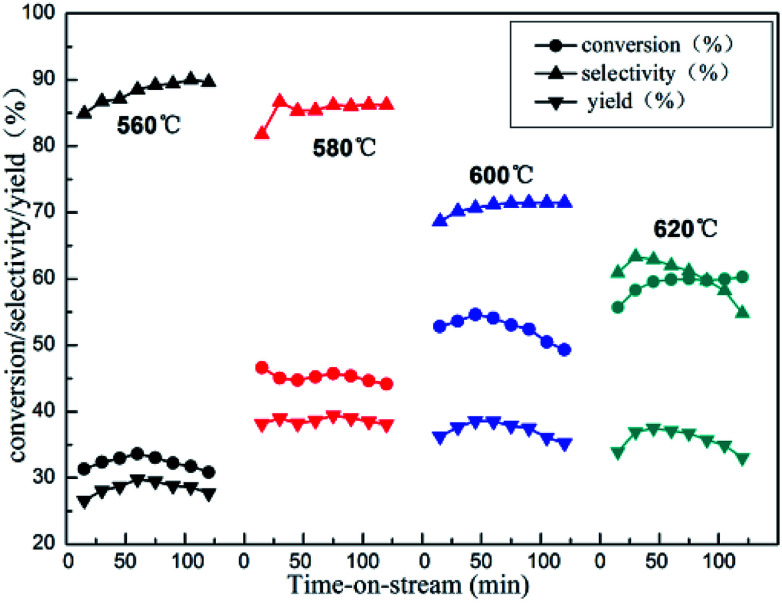
The reactivity of isobutane dehydrogenation over the 10%Zn/Al_2_O_3_ catalyst. Reaction condition: GHSV = 300 h^−1^.

Isobutane dehydrogenation is an endothermic reaction in thermodynamics, which need relatively high temperature to obtain excellent isobutene yield. The effect of reaction temperature on catalytic activity of 10%Zn/Al_2_O_3_ catalyst was investigated in Fig. 8. The conversion of isobutane was 45.0% and the selectivity of isobutene was 86.7% at 580 °C after 30 min. With the increase of the reaction temperature, the conversion of isobutane obviously increased and selectivity of isobutene significantly decreased, which was quite conformed to the characteristic of alkane dehydrogenation reaction. However, the isobutane conversion dropped drastically, isobutene selectivity just increased slightly with the reaction temperature decreased to 560 °C. In view of catalytic stability, 580 °C was deemed as the optimal reaction temperature.

**Fig. 9:**
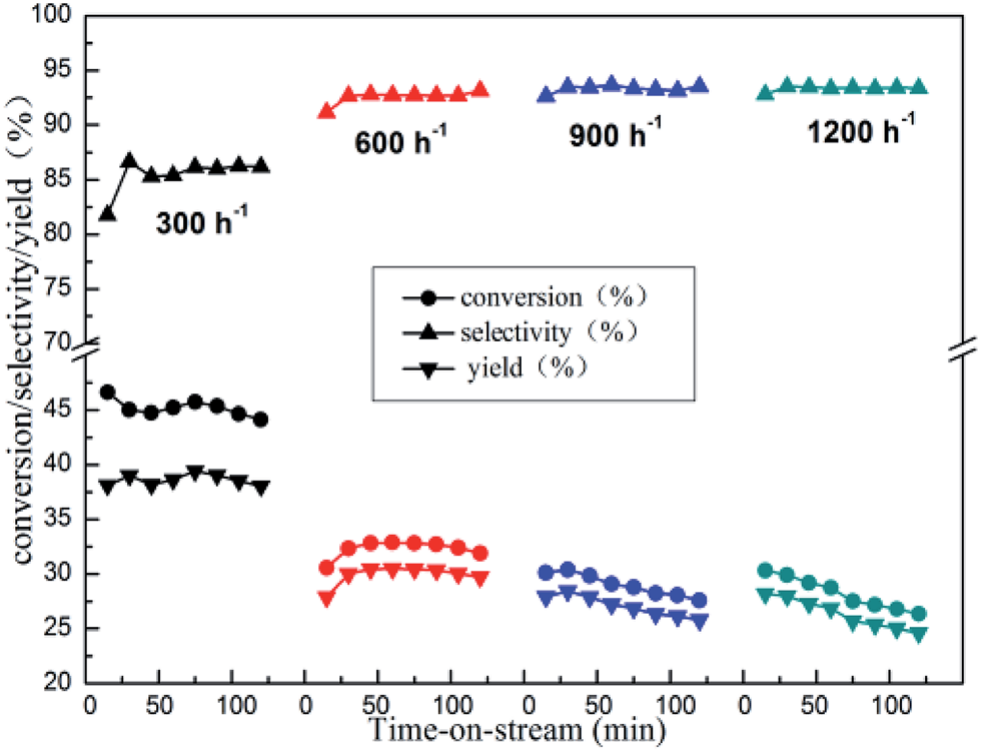
The reactivity of isobutane dehydrogenation over the 10%Zn/Al_2_O_3_ catalyst. Reaction condition: *T* = 580 °C.

The impact of the GHSV on catalytic activity was also carefully studied over 10%Zn/Al_2_O_3_ catalyst (Fig. 9). As we can see, with GHSV increasing from 300 to 600 h^−1^, the initial isobutane conversion decreased obviously (from 46.6% to 30.6%), while initial isobutene selectivity increased (from 81.8% to 91.1%). As the GHSV continues to rise, it became slope that isobutane conversion decreased with time on stream.

**Fig. 10:**
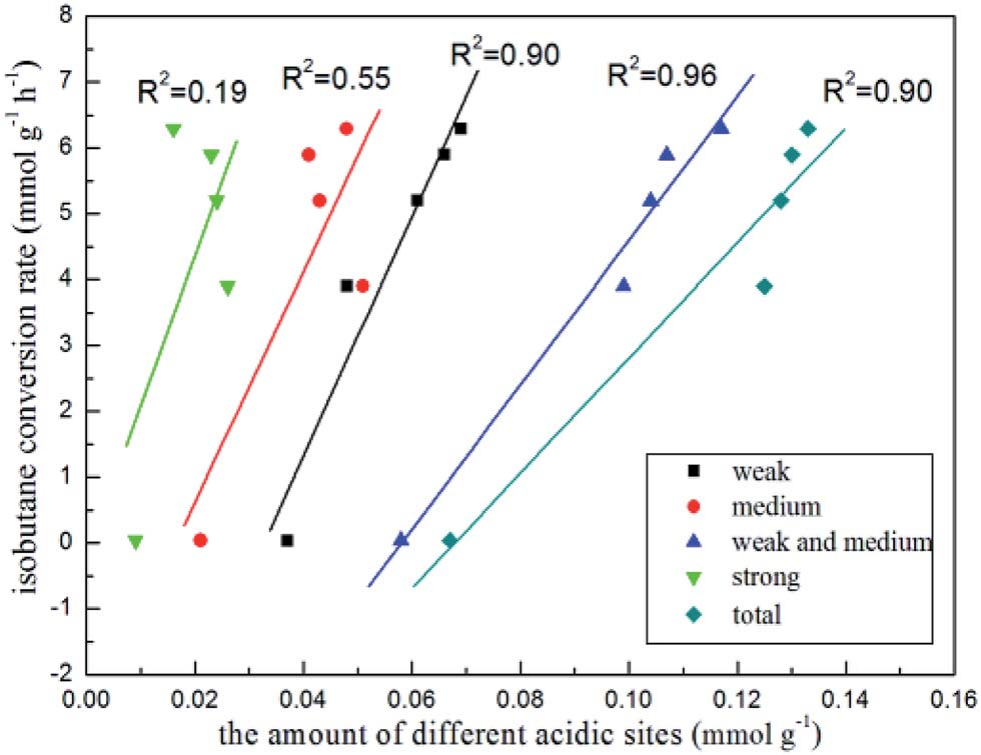
The trend of isobutane conversion rate with the amount of different kinds of the acidic sites.

In order to further investigate what were active sites for isobutane dehydrogenation, we tested this reaction at 580 °C over ordered mesoporous Al_2_O_3_ and commercial ZnO as a contrast (Fig. S3†). It can be seen that initial isobutane conversion and initial isobutene yield over Al_2_O_3_ were only 3.1% and 2.1%, which illustrated that ordered mesoporous Al_2_O_3_ was inactive to the reaction. ZnO exhibited slightly higher initial dehydrogenation activity (9.2% isobutane conversion and 5.5% isobutene yield). It seemed that bulk ZnO particle had some catalytic ability. Surprisingly, with the introduction of Zn, the *x*Zn/Al_2_O_3_ catalysts exhibited very excellent initial catalytic activity, even then the ordered mesopore collapsed and Zn species was not highly dispersed over 15%Zn/Al_2_O_3_ catalyst. Considering the possibility of zinc incorporated into Al_2_O_3_ framework over 15%Zn/Al_2_O_3_ catalyst, we deduced that the existence of framework zinc may play a crucial role. However, there is no direct evidence for this. The essential role of Zn species of *x*Zn/Al_2_O_3_ in isobutane dehydrogenation still need to be identified, which is the focus of the following work in our laboratory. In addition, it is well-known that the amount of different acidic sites of catalyst are the key to affect catalytic activity in alkane dehydrogenation reaction.^37^ The isobutane conversion rate *vs.* the number of different acidic sites on the basis of Table 2 were plotted (Fig. 10). The isobutane conversion rate *versus* the amount of strong acidic sites showed the worst linear correlation (*R*^2^ = 0.19), nevertheless, *versus* the amount of weak and medium acidic sites presented the best linear correlation (*R*^2^ = 0.96), indicating that strong acidic sites were not the decisive factor for isobutane conversion, while both the weak and medium acidic sites took the important part in isobutane conversion. Besides, the isobutane selectivity was obviously increased with the decrease of the amount of strong acidic sites over 3–10%Zn/Al_2_O_3_ catalysts, indicated strong acid sites mainly catalyzed side reactions.

### 3.8. The stability of ordered mesoporous Zn/Al_2_O_3_ catalyst

#### 3.8.1. XRD analysis

The wide-angle XRD patterns of the spent catalysts are shown in Fig. 1. It was observed that there is no difference between fresh and spent catalysts. The crystalline ZnO phase was also visible over spent 15%Zn/Al_2_O_3_ catalyst, and no Zn species over the spent 10%Zn/Al_2_O_3_ catalyst as well, indicating that Zn species were stable without phase transformation during this dehydrogenation reaction.

#### 3.8.2. Nitrogen adsorption–desorption analysis

The nitrogen adsorption–desorption analysis of the spent and regenerated catalysts are in Fig. S4.† Compared with the corresponding fresh catalysts, the isotherms of spent catalysts had no obvious changed and still presented uniform pore size. The textural properties are presented in Table 1. It was clearly seen that the BET specific surface areas of the spent 10%Zn/Al_2_O_3_ and 15%Zn/Al_2_O_3_ catalysts were greatly reduced to 149.7 m^2^ g^−1^ and 25.2 m^2^ g^−1^, respectively. It was probably due to that coke deposition on the spent catalyst blocked a portion of the pore, thus leading to the decrease of textural performance. After five dehydrogenation–regeneration cycles, the slightly diminished specific surface area (163.3 → 158.6 m^2^ g^−1^) and pore volume (0.43 → 0.42 cm^3^ g^−1^) of the regenerated 10%Zn/Al_2_O_3_ demonstrated the formed coke can be eliminated by regeneration process.

#### 3.8.3. TG-DSC analysis

**Fig. 11:**
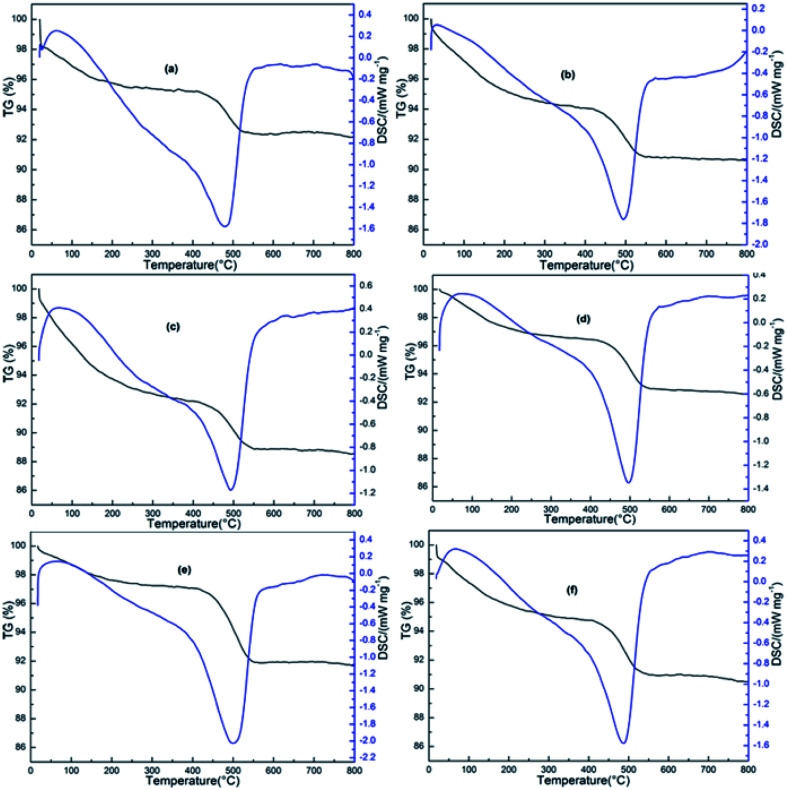
TG-DSC curves of the spent catalysts at 580 °C reaction: (a) 3%Zn/Al_2_O_3_; (b) 5%Zn/Al_2_O_3_; (c) 7%Zn/Al_2_O_3_, (d) 10%Zn/Al_2_O_3_; (e) 15%Zn/Al_2_O_3_ and the spent catalyst at 620 °C reaction: (f) 10%Zn/Al_2_O_3_.

As we all know, coking is usually the main reason for catalyst deactivation in alkane dehydrogenation reaction. TG-DSC is a very efficient technique to analyze the coke amount and the characterization results are shown in Fig. 11. The TG curves presented downtrend with the increase of temperature, which could be divided into two stages. In the first stage, the weight losses in TG curves up to 300 °C, ascribed to the loss of physically adsorbed water and impurities. The second stage, located in the 350–600 °C, the losses was assigned to the removal of coke. The coke amount in the spent 3%Zn/Al_2_O_3_, 5%Zn/Al_2_O_3_, 7%Zn/Al_2_O_3_ and 10%Zn/Al_2_O_3_ were 2.97%, 3.22%, 3.29% and 3.45%, respectively. Which similar coke amount was consistent with the same good stability of these ordered mesoporous catalysts. However, with increasing Zn content to 15%, coke amount was markedly increased (5.19%). Combined with the poor stability of 15%Zn/Al_2_O_3_ catalyst, the reason of serious deactivation may caused by more coke, which also indicated ordered meso-structure could effectively inhibited the formation of coke.^38^ Moreover, during 620 °C reaction temperature, the coke amount in the spent 10%Zn/Al_2_O_3_ catalyst increased to 3.79%. It explained the stability decreased with the increase of reaction temperature.

#### 3.8.4. The dehydrogenation–regeneration cycles

**Fig. 12:**
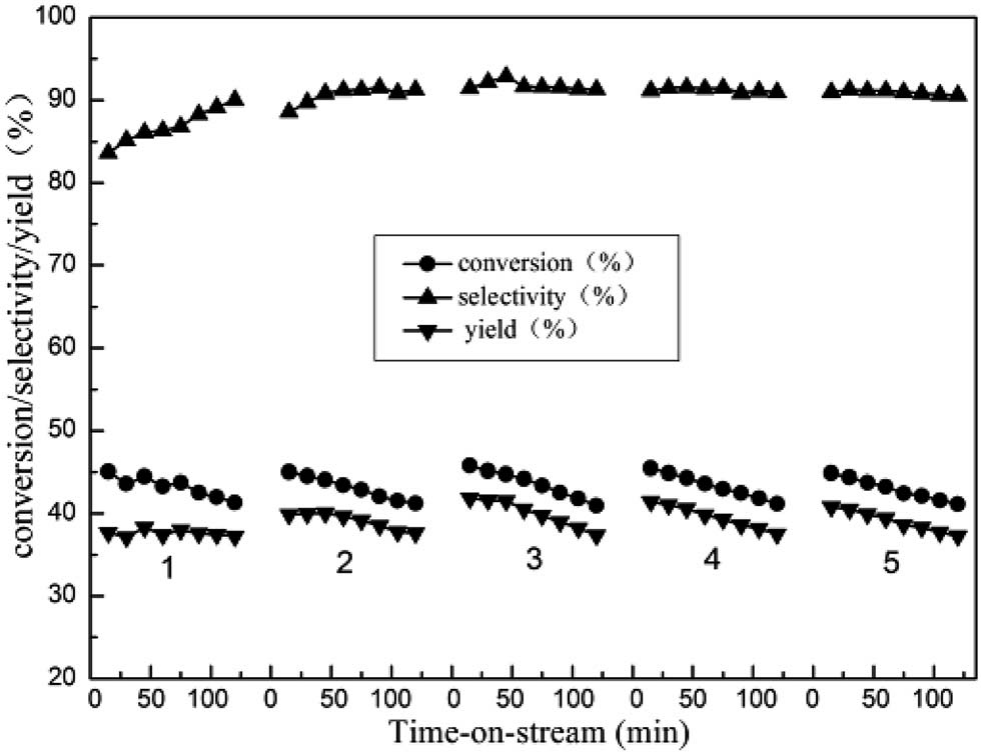
The five dehydrogenation–regeneration cycles of isobutane dehydrogenation over 10%Zn/Al_2_O_3_ catalyst. Reaction condition: *T* = 580 °C, GHSV = 300 h^−1^.

In order to examine the regenerative ability of the 10%Zn/Al_2_O_3_ catalyst, the five dehydrogenation–regeneration cycles were investigated (Fig. 12). By calculation, the initial isobutane conversion, isobutene selectivity and yield were 45.1%, 83.6% and 37.7% in the first cycle, respectively. With increasing reaction time, the catalytic activity decreased gradually. Following by 2 h regeneration of catalyst at 600 °C in air, the activity of catalyst was obviously restored, which affirmed coke was the main reason of catalyst deactivation. In the five cycle, the initial isobutane conversion, isobutene selectivity and yield were decrease slightly, indicated the high regenerative ability of the catalyst.

## 4. Conclusions

A series of *x*Zn/Al_2_O_3_ materials with various Zn content were simply prepared *via* one pot EISA strategy and tested in isobutane dehydrogenation reaction. The obtained materials with Zn content up to 10% possessed well-ordered mesopore with large specific surface areas, big pore volumes and uniform pore size. Zinc species in these catalysts was highly dispersed on support surface or incorporated into framework, while ZnO crystal particles were observed with 23.8 nm size in the case of 15%Zn. It was found that these materials presented excellent catalytic activity. Note that the total acidic sites gradually increased, while the strong acidic sites decreased slowly with the increase of Zn content until 10%. The decrease of strong acid sites is conducive to the promotion of isobutene selectivity and the weak and medium acidic sites played a role in isobutane conversion. In addition, the catalyst exhibited excellent stability and high regenerative ability, which demonstrated potential for commercial applications.

## Conflicts of interest

There are no conflicts to declare.

## Supplementary Material

RA-009-C9RA00217K-s001
