# Supplementary material for: Facile synthesis of ordered mesoporous zinc alumina catalysts and their dehydrogenation behavior
Source: RSC Adv. 2019 Mar 28;9(17):9828–37. doi: 10.1039/c9ra00217k (PMC9062119; doi:10.1039/c9ra00217k)
Supplement: RA-009-C9RA00217K-s001 [file RA-009-C9RA00217K-s001.pdf]

Electronic Supplementary Material (ESI) for RSC Advances.
This journal is © The Royal Society of Chemistry 2019

Supporting Information

Facile synthesis of ordered mesoporous zinc alumina catalysts and dehydrogenation behavior

Ming Cheng [a,b], Huahua Zhao [a], Jian Yang [a], Jun Zhao [a] , Liang Yan [a], Huanling Song [a,*], Lingjun Chou [a,c,*]

[a] State Key Laboratory for Oxo Synthesis and Selective Oxidation, Lanzhou Institute of Chemical Physics (LICP), Chinese Academy of Sciences, Lanzhou 730000, PR China

[b] University of Chinese Academy of Sciences, Beijing 100049, PR China

[c] Suzhou Research Institute of LICP, Chinese Academy of Sciences, Suzhou 215123, PR China

*Corresponding author. *E-mail address*: ljchou@licp.cas.cn (Lingjun Chou), Tel: +86 931 4968 066, Fax: +86 931 4968 129; songhl@licp.cas.cn(Huanling Song), Tel: +86 931 4968 066, Fax: +86 931 4968 129;

S1. NH_3-TPD analysis

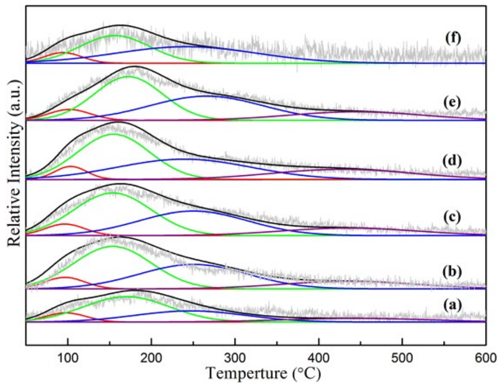

Fig. S1. The NH_3-TPD profiles of the as-synthesized xZn/Al_2O_3 catalysts and Al_2O_3: (a) Al_2O_3; (b) 3%Zn/Al_2O_3; (c) 5%Zn/Al_2O_3; (d) 7%Zn/Al_2O_3; (e) 10%Zn/Al_2O_3; (f) 15%Zn/Al_2O_3.

S2. NH_3-TPD profiles

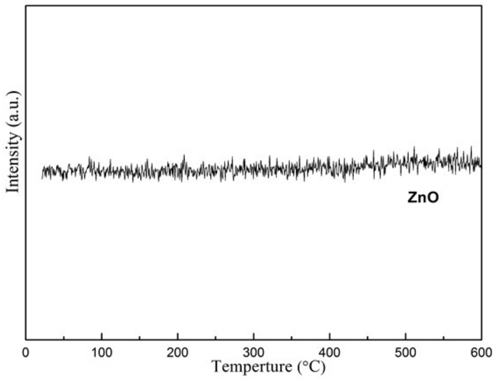

Fig. S2. The NH_3-TPD profile of ZnO.

S3. The catalytic dehydrogenation of isobutane over the ordered mesoporous Al_2O_3 and commercial ZnO.

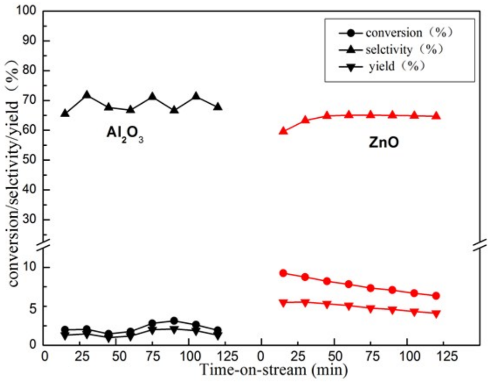

Fig. S3. The catalytic dehydrogenation of isobutane over the ordered mesoporous Al_2O_3 and commercial ZnO. Reaction condition: T = 580 °C, GHSV = 300 h^{-1}.

S4. Nitrogen adsorption–desorption analysis

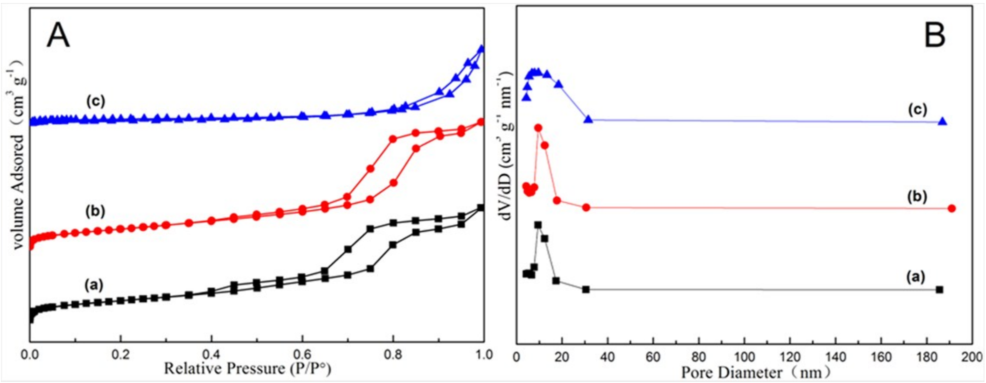

Fig. S4. The nitrogen adsorption–desorption analysis of the catalysts: (a) the spent10%Zn/Al_2O_3; (b) the fifth regenerated 10%Zn/Al_2O_3; (c) the spent 15%Zn/Al_2O_3.
